# PKM2 regulates osteoclastogenesis by affecting osteoclast precursor cell fusion *via* downregulation of OC-STAMP and DC-STAMP

**DOI:** 10.1016/j.jbc.2025.108439

**Published:** 2025-03-22

**Authors:** Cong Cai, Jiawei Jiang, Song Li, Chenghao Gao, Hongxu Pu, Libo Zhao, Jun Xiao

**Affiliations:** 1 Department of Orthopedics, Tongji Hospital, Tongji Medical College, Huazhong University of Science and Technology, Wuhan, China; 2 Department of Traumatic Surgery, Tongji Hospital, Tongji Medical College, Huazhong University of Science and Technology, Wuhan, China

**Keywords:** PKM2, osteoporosis, osteoclast, activator, OC-STAMP, DC-STAMP

## Abstract

Osteoporosis is a common bone disease that has become a serious public health problem with the aging of population. Osteoclasts are the only cells in body that can resorb bone, whose dysfunction is closely related to osteoporosis. Pyruvate kinase M2 (PKM2) is one of the essential rate-limiting enzymes in the process of glycolysis. This study aimed to elucidate the role of PKM2 in osteoclastogenesis and bone resorption. Bone marrow–derived macrophages were transfected with adenovirus to knock down the expression of PKM2 gene or treated with the PKM2 activators, DASA-58 and TEPP-46. Osteoclast formation was detected by tartrate-resistant acid phosphatase staining, osteoclast-specific gene and protein expression was detected by RT–quantitative PCR and Western blotting, and the effect of DASA-58 on osteoclast gene expression at the transcriptional level was examined by RNA sequencing. The results showed that knockdown of PKM2 by adenoviral transfection or treatment with PKM2 activators, DASA-58 and TEPP-46, inhibited osteoclast differentiation and suppressed the expression of osteoclast-associated genes in bone marrow–derived macrophages. Furthermore, PKM2 activators, DASA-58 and TEPP-46, could inhibit several signaling pathways in osteoclasts; knockdown of PKM2 or treatment with PKM2 activators, DASA-58 and TEPP-46, both affected osteoclast precursor cell fusion by inhibiting the expression of osteoclast stimulatory transmembrane protein (OC-STAMP) and dendritic cell–specific transmembrane protein (DC-STAMP). Therefore, PKM2 is closely related to osteoclast differentiation and formation, and the development of new therapeutic strategies targeting the PKM2 gene in osteoclasts may be feasible for the prevention and treatment of osteoporosis.

Osteoporosis is a systemic bone disease characterized mainly by decreased bone mass and deterioration of bone tissue microarchitecture, which increases bone fragility and susceptibility to fracture. With the aging of population, osteoporosis-related fracture has been the leading cause of disability and mortality in the elderly and a major public health problem with increasing medical and socioeconomic burden (1, 2).

Bone is constantly remodeling to maintain a dynamic balance. Bone remodeling is a physiological process in which old and damaged bone is removed by osteoclasts and replaced by new bone formed by osteoblasts. Normally, osteoclasts and osteoblasts work in concert to maintain the balance of bone tissue (3). Osteoclasts are the only cells in body that perform the function of bone resorption, and their abnormally increased activity in pathological conditions disturbs the balance of bone remodeling and would lead to bone metabolic diseases, most commonly osteoporosis (4).

Osteoclasts are tissue-specific macrophage polysomes formed by the differentiated mononuclear phagocyte precursor cells located on or near the bone surface. Induced by cytokines such as macrophage colony-stimulating factor (M-CSF) and receptor activator of NF-κB ligand (RANKL), several signaling pathways are activated in mononuclear phagocytes, including NF-κB, mitogen-activated protein kinases (MAPKs) (extracellular signal–regulated kinase [ERK], c-Jun NH2-terminal kinase [JNK], and p38), and PI3K–AKT. Mononuclear phagocytes then progressively differentiate and fuse step by step to form mature osteoclasts, expressing several specific genes, such as tartrate-resistant acid phosphatase (TRAP), cathepsin K (CTSK), and nuclear factor of activated T cells 1 (NFATc1) to play a role in bone resorption (5).

Pyruvate kinase (PK) is one of the key rate-limiting enzymes in glycolysis (6, 7). PK regulates the final step of glycolysis, converting phosphoenolpyruvate to pyruvate and phosphorylating ADP to ATP. It consists of four isozymes: PKL, PKR, PKM1, and PKM2 (7), of which PKM2 exists mainly as a monomer or a dimer with low enzymatic activity and acts as a transcriptional coactivator that regulates the activity of various transcription factors by translocating to the nucleus (8, 9, 10). PKM2-mediated glycolysis has been shown to regulate the growth, invasion, and osteoclast formation of odontogenic keratocyst fibroblasts (11). In RAW264.7 cells, hypoxia or hypoxia-inducible factor-1α enhanced osteoclast activation and acid secretion by regulating the glycolytic enzymes, lactate dehydrogenase A, glucose kinase, PKM2, and phosphofructokinase 1 (12, 13, 14). In addition, PKM2 inhibits osteogenic differentiation and promotes lipogenic differentiation of bone marrow mesenchymal stem cells by regulating the β-catenin signaling pathway and mitochondrial fusion and fission (15, 16). In contrast, TRAF4 negatively regulates adipogenesis of bone marrow mesenchymal stem cells by activating PKM2 activity (17). It has been shown that high expression of PKM2 in tissues of osteosarcoma patients is associated with poor prognosis, and knockdown of PKM2 can inhibit proliferation and invasion of osteosarcoma cells *in vitro* and *in vivo* (18, 19, 20). The aforementioned studies suggest that PKM2 may play an important role in bone metabolic homeostasis.

Bisphosphonates and anti-RANKL antibodies are currently the first-line therapeutic agents targeting the excessive osteoclast activity (21). However, because of the close relationship between osteoclasts and osteoblasts, inhibition of osteoclasts by bisphosphonates or anti-RANKL antibodies will inevitably affect bone formation. Meanwhile, side effects, complications, and lack of long-term adherence are also the limitations of these agents (22). Therefore, new therapeutic strategies targeting osteoclast metabolism are needed for the prevention and treatment of osteoporosis. In the present study, we investigated the effect of PKM2 on osteoclast formation and function and studied the mechanism involved.

## Results

### Knockdown of PKM2 inhibited osteoclast differentiation and the expression of osteoclast-associated genes

**Figure 1:**
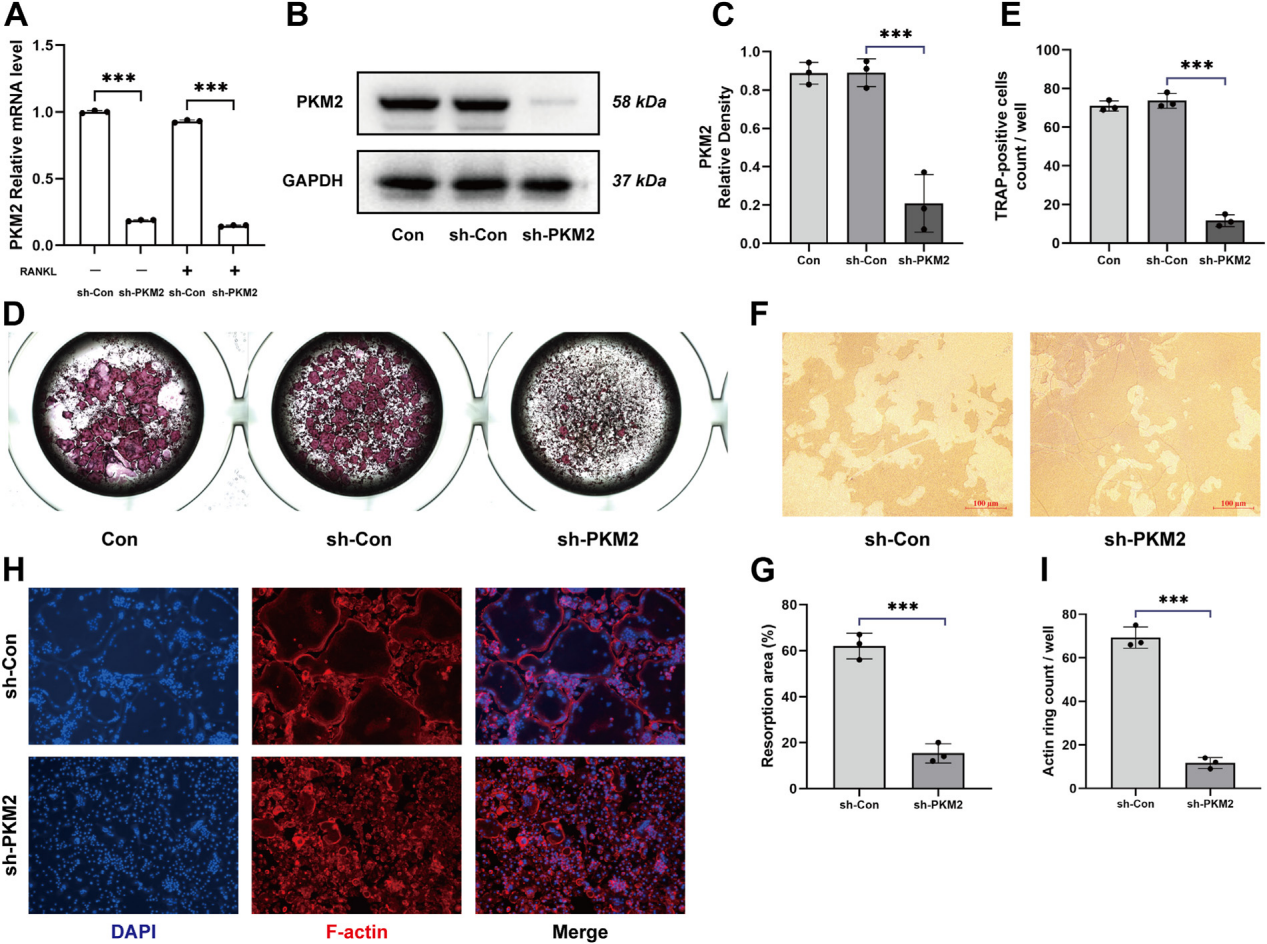
**Knockdown of PKM2 inhibits osteoclast differentiation of BMMs.** BMMs were infected with an adenovirus carrying PKM2-specific shRNA or a control adenovirus to verify the expression of PKM2 mRNA (*A*) and protein (*B*) in the cells. *C*, relative quantification of PKM2 protein. Knockdown of PKM2 inhibited osteoclast differentiation (*D*) and bone resorption function (*F*) of BMMs. *E*, number of TRAP-positive cells. *G*, quantification of bone resorption area. Cells were intervened and cultured for 7 days, cell morphology was observed (*H*), and number of actin rings is counted (*I*). Data are expressed as the mean ± SD of three independent replicates. ∗*p* < 0.05, ∗∗*p* < 0.01, and ∗∗∗*p* < 0.001. BMM, bone marrow–derived macrophage; PKM2, pyruvate kinase M2; TRAP, tartrate-resistant acid phosphatase.

To investigate the role of PKM2 in osteoclast differentiation, bone marrow–derived macrophages (BMMs) were infected with adenovirus carrying PKM2-specific shRNA or control adenovirus. Figure 1, *A*–*C* showed that PKM2 mRNA and protein expression levels were significantly decreased by adenovirus carrying PKM2 shRNA compared with the control group. The transfected BMMs were then induced with RANKL, and our study results showed that TRAP expression was significantly reduced in the sh-PKM2 group compared with the sh-Con group, indicating that knockdown of PKM2 inhibited BMM differentiation into osteoclasts (Fig. 1, *D*–*G*). Large and round F-actin rings of BMMs were observed in the control group, and knockdown of PKM2 obviously affected the formation of F-actin rings, suggesting that knockdown of PKM2 suppressed osteoclast bone resorption (Fig. 1, *H* and *I*).

**Figure 2:**
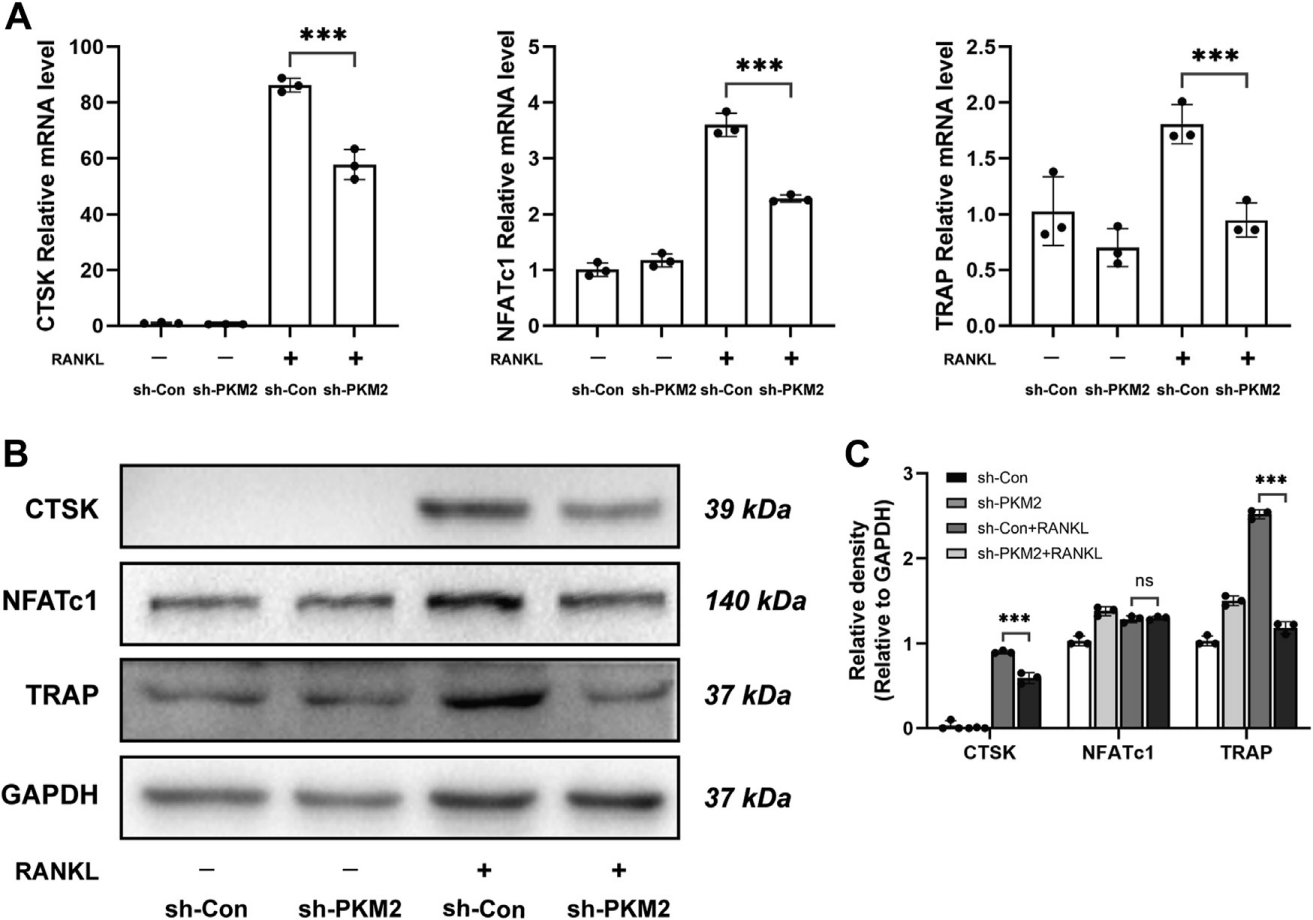
**Knockdown of PKM2 inhibits the expression of osteoclast-related genes and proteins.** The expression of mRNA (*A*) and protein (*B*) of osteoclast-related genes, CTSK, NFATc1, and TRAP, in BMMs was inhibited after the knockdown of PKM2. *C*, relative quantification of CTSK, NFATc1 and TRAP protein. Data are displayed as the mean ± SD of three independent replicates. ∗*p* < 0.05, ∗∗*p* < 0.01, ∗∗∗*p* < 0.001. BMM, bone marrow–derived macrophage; CTSK, cathepsin K; NFATc1, nuclear factor of activated T cell 1; PKM2, pyruvate kinase M2; TRAP, tartrate-resistant acid phosphatase.

In addition, to further investigate the effect of PKM2 on osteoclast activity, PKM2 was knocked down in BMMs and osteoclast differentiation was induced with RANKL. We examined the expression levels of the osteoclast-associated genes, such as CTSK, NFATc1, and TRAP, and found that the mRNA and protein levels of CTSK, NFATc1, and TRAP were decreased (Fig. 2, *A*–*C*), suggesting that PKM2 played an essential role in osteoclast differentiation and formation.

### PKM2 activators, DASA-58 and TEPP-46, inhibited osteoclast formation

**Figure 3:**
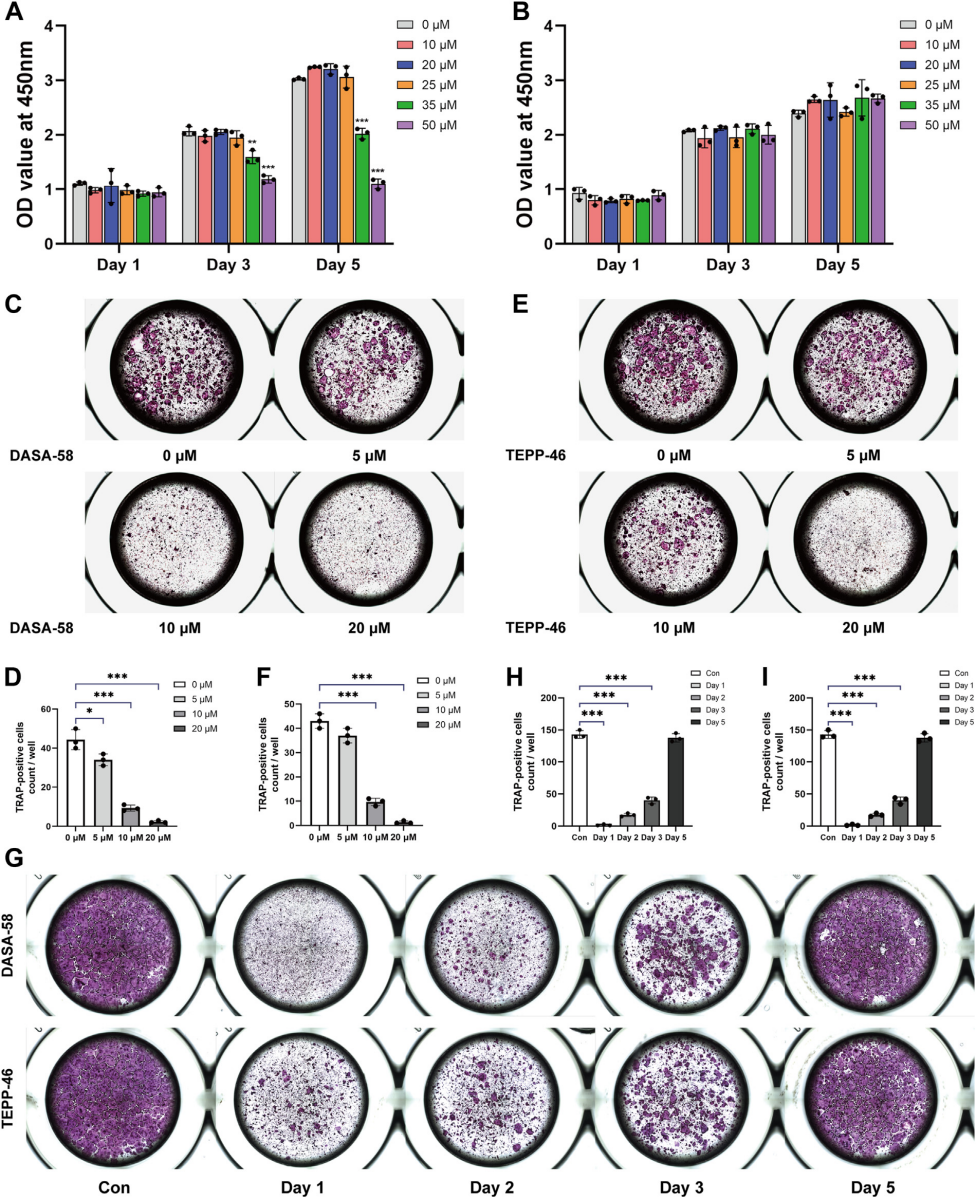
**PKM2 activators, DASA-58 and TEPP-46, inhibit osteoclast differentiation of BMMs.** BMMs were treated with different concentrations of PKM2 activators DASA-58 (*A*) and TEPP-46 (*B*), respectively, and MTT assays showed their effect on cell proliferation. The TRAP staining showed that osteoclast differentiation of BMMs was inhibited by treatment with appropriate concentrations of PKM2 activators DASA-58 (*C*) and TEPP-46 (*E*), respectively. Number of TRAP-positive cells by treatment with appropriate concentrations of DASA-58 (*D*) and TEPP-46 (*F*). *G*, the representative TRAP staining images of BMMs after treating with PKM2 activators at different time points. Number of TRAP-positive cells after treating with DASA-58 (*H*) and TEPP-46 (*I*) at different time points. Data are expressed as the mean ± SD of three independent replicates. ∗*p* < 0.05, ∗∗*p* < 0.01, and ∗∗∗*p* < 0.001. BMM, bone marrow–derived macrophage; PKM2, pyruvate kinase M2; TRAP, tartrate-resistant acid phosphatase.

BMMs were treated with different concentrations of PKM2 activators, TEPP-46 or DASA-58, respectively, and induced with RANKL to differentiate toward osteoclasts. The PKM2 activators, DASA-58 and TEPP-46, were able to promote PKM2 tetramer formation and inhibit PKM2 entry into the nucleus. Both DASA-58 and TEPP-46 were found to inhibit osteoclast formation in a concentration-dependent manner (Fig. 3, *A*–*F*). Similar results were obtained after treatment with DASA-58 and TEPP-46 at different time points of osteoclast differentiation (Fig. 3, *G*–*I*).

**Figure 4:**
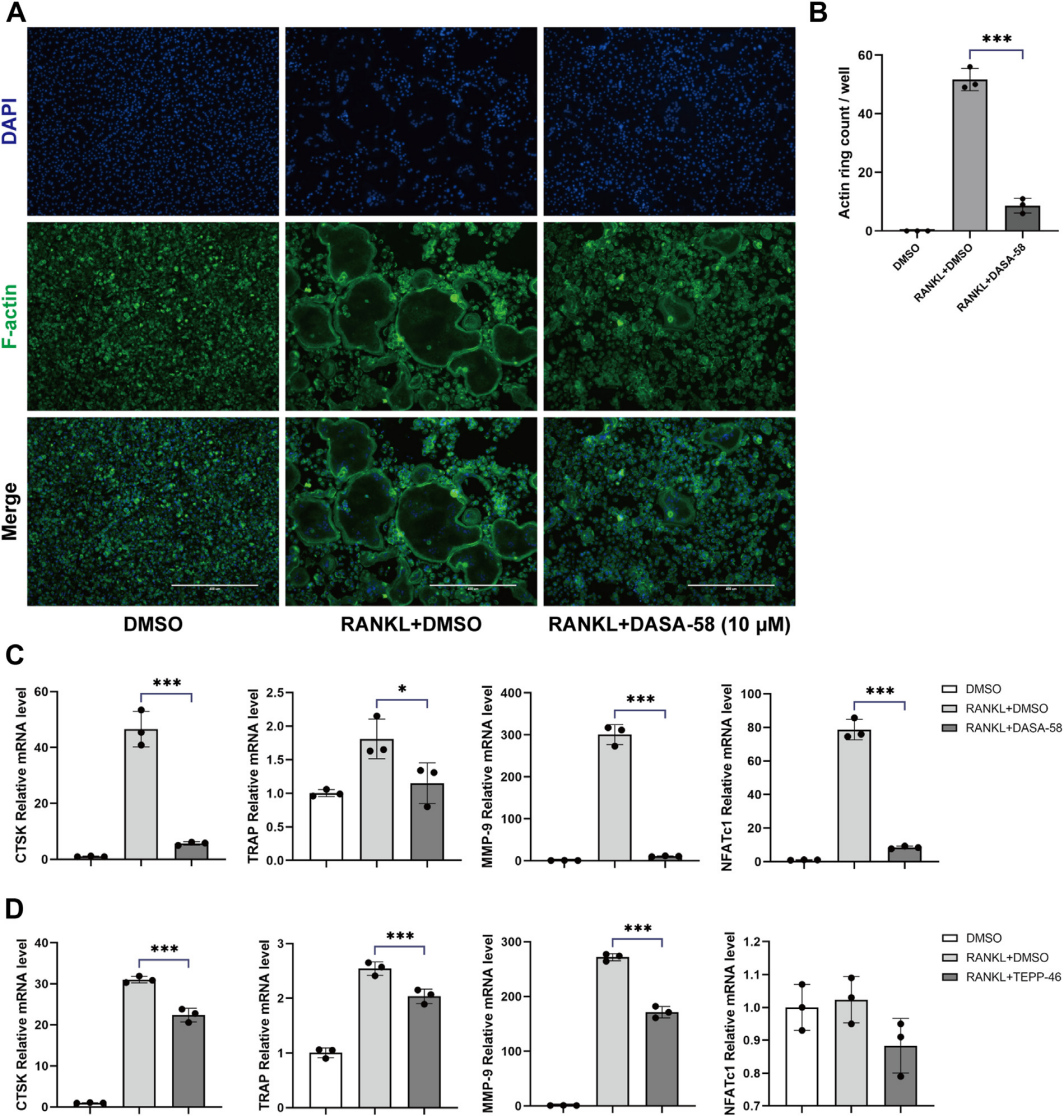
**PKM2 activators, DASA-58 and TEPP-46, inhibit osteoclast-related gene expression.***A* and *B*, osteoclast differentiation was inhibited when cells were treated with PKM2 activator DASA-58. *A*, cells were intervened and cultured for 7 days, and cell morphology was observed. *B*, number of actin rings by treatment with DASA-58 (10 μM). The expression of osteoclast-associated genes, CTSK, NFATc1, TRAP, and MMP-9, was inhibited after the addition of PKM2 activators DASA-58 (*C*) and TEPP-46 (*D*). DASA-58 and TEPP-46 were used at 10 μM in the culture medium. Data are displayed as the mean ± SD of three independent replicates. ∗*p* < 0.05, ∗∗*p* < 0.01, and ∗∗∗*p* < 0.001. BMM, bone marrow–derived macrophage; CTSK, cathepsin K; NFATc1, nuclear factor of activated T cell 1; PKM2, pyruvate kinase M2; TRAP, tartrate-resistant acid phosphatase.

We further investigated the role of PKM2 activators, DASA-58 and TEPP-46, in osteoclasts. BMMs were treated with appropriate concentrations of the PKM2 activators, DASA-58 and TEPP-46, respectively, and induced with RANKL. F-actin rings were found to be smaller and more irregular in the RANKL+DASA-58 group when compared with the RANKL + dimethyl sulfoxide (DMSO) group by immunofluorescence detection (Fig. 4, *A* and *B*). RNA and protein were extracted, and the results of RT–quantitative PCR (qPCR) and Western blotting assays showed that DASA-58 and TEPP-46 also inhibited the expression levels of mRNA and protein of osteoclast-related genes, such as CTSK, NFATc1, and TRAP (Fig. 4, *C* and *D*).

### PKM2 activators, DASA-58 and TEPP-46, downregulated several signaling pathways in RANKL-treated BMMs

**Figure 5:**
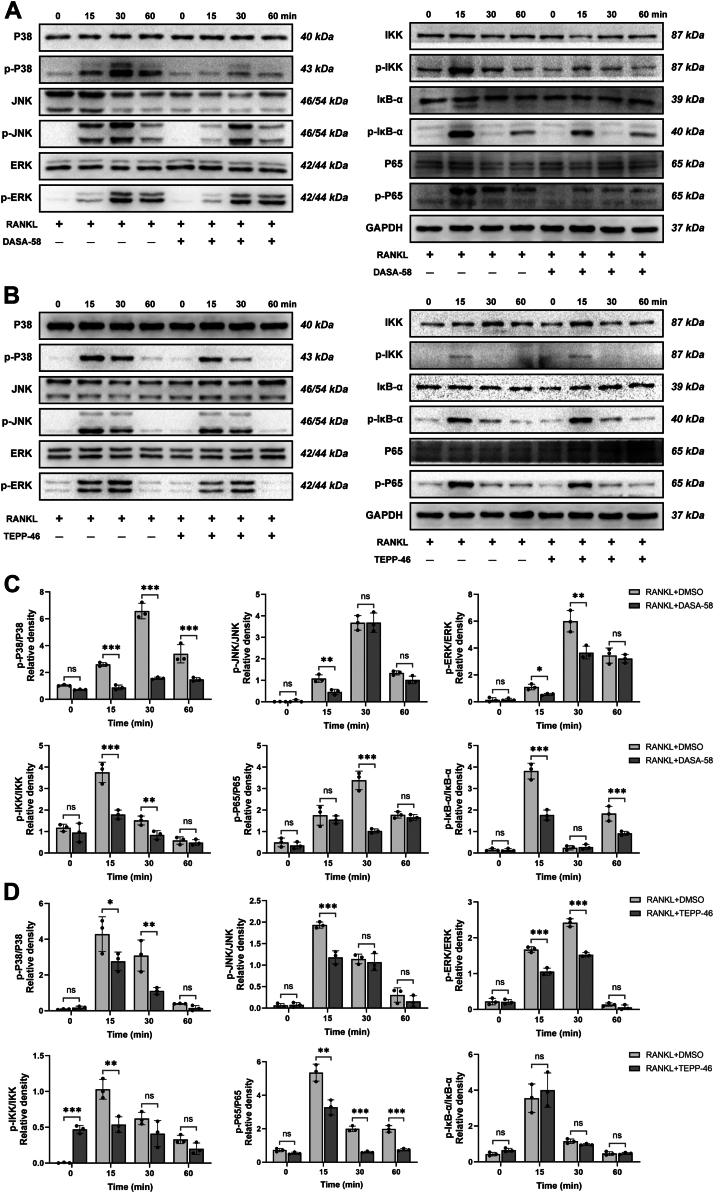
**PKM2 activators, DASA-58 and TEPP-46, inhibit multiple signaling pathways in BMMs.** BMMs were treated with PKM2 activators DASA-58 (*A*) and TEPP-46 (*B*), respectively, in parallel with RANKL induction, and proteins were extracted at the corresponding time points, showing their effects on P38, JNK, ERK, P65, IκB-α, and IKKα/β protein phosphorylation. DASA-58 and TEPP-46 were used at 10 mM in the culture medium. Relative quantification of P38, JNK, ERK, P65, IκB-α, and IKKα/β protein phosphorylation with PKM2 activators DASA-58 (*C*) and TEPP-46 (*D*). Data are expressed as the mean ± SD of three independent replicates. ∗*p* < 0.05, ∗∗*p* < 0.01, and ∗∗∗*p* < 0.001. BMM, bone marrow–derived macrophage; ERK, extracellular signal–regulated kinase; JNK, c-Jun NH2-terminal kinase; PKM2, pyruvate kinase 2; RANKL, receptor activator of nuclear factor-κB ligand.

To investigate the molecular mechanism by which the PKM2 activators, DASA-58 and TEPP-46, inhibited osteoclast differentiation, BMMs were treated with appropriate concentrations of DASA-58 and TEPP-46, respectively, and induced with RANKL, and proteins were extracted at the appropriate time points. Western blotting assay showed that DASA-58 and TEPP-46 could inhibit the phosphorylation of several protein pathways (Fig. 5), indicating that the PKM2 activators, DASA-58 and TEPP-46, could affect osteoclast differentiation and formation by inhibiting the activation of NF-κB and MAPK pathways.

### PKM2 regulated osteoclastogenesis *via* inhibiting the expression of OC-STAMP and DC-STAMP

**Figure 6:**
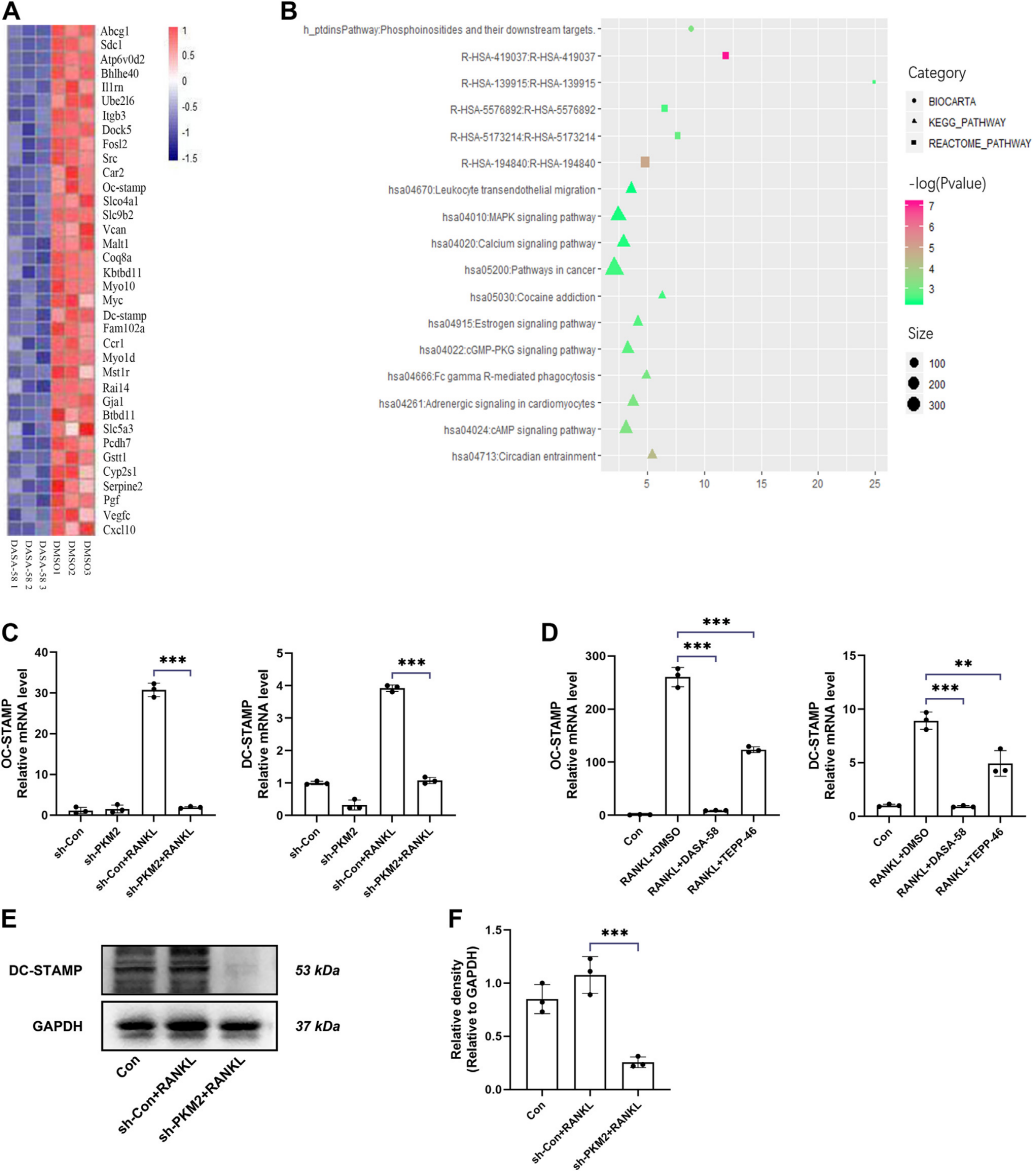
**PKM2 regulated osteoclastogenesis *via* inhibiting the expression of OC-STAMP and DC-STAMP.***A*, heatmap of part of the cluster analysis results of differentially expressed genes (threshold: log fold change <−1, *p* < 0.05). *B*, gene set enrichment analysis was used to select the involved pathways. *C*, OC-STAMP and DC-STAMP mRNA expression was inhibited after the knockdown of PKM2. *D*, OC-STAMP and DC-STAMP mRNA expression was suppressed after treatment with PKM2 activators, DASA-58 and TEPP-46. *E*, DC-STAMP protein expression was inhibited after the knockdown of PKM2. *F*, relative quantification of DC-STAMP protein. Data are displayed as the mean ± SD of three independent replicates. ∗*p* < 0.05, ∗∗*p* < 0.01, and ∗∗∗*p* < 0.001. DC-STAMP, dendritic cell–specific transmembrane protein; OC-STAMP, osteoclast stimulatory transmembrane protein; PKM2, pyruvate kinase 2.

The effect of PKM2 on osteoclast differentiation was further investigated at the transcriptional level using RNA sequencing (RNA-seq). BMMs were treated with appropriate concentrations of DASA-58 and an equal volume of DMSO, induced with RANKL for 3 days, and the RNA was extracted for RNA-seq. Figure 6*A* showed part of the cluster analysis results of differentially expressed genes and indicated that PKM2 activator DASA-58 could inhibit the expression of osteoclast-related genes in RANKL-treated BMMs at the transcriptional level. Gene set enrichment analysis showed that several signaling pathways were affected, the most significant of which was the MAPK pathway (Fig. 6*B*).

To verify whether osteoclast stimulatory transmembrane protein (OC-STAMP) and dendritic cell–specific transmembrane protein (DC-STAMP) had an essential role in the regulation of PKM2 in osteoclastogenesis, BMMs were treated with PKM2 activators, DASA-58 and TEPP-46, respectively and induced with RANKL. Total RNA was extracted at specific time points for RT–qPCR, and the results showed that the expression of OC-STAMP and DC-STAMP was inhibited (Fig. 6, *C* and *D*), suggesting that the PKM2 activators, DASA-58 and TEPP-46, could inhibit cell fusion by suppressing the expression of OC-STAMP and DC-STAMP. DC-STAMP protein expression was detected and quantified, which showed that DC-STAMP protein decreased in the sh-PKM2 group, also indicating that DC-STAMP expression was inhibited after the knockdown of PKM2 (Fig. 6, *E* and *F*).

**Figure 7:**
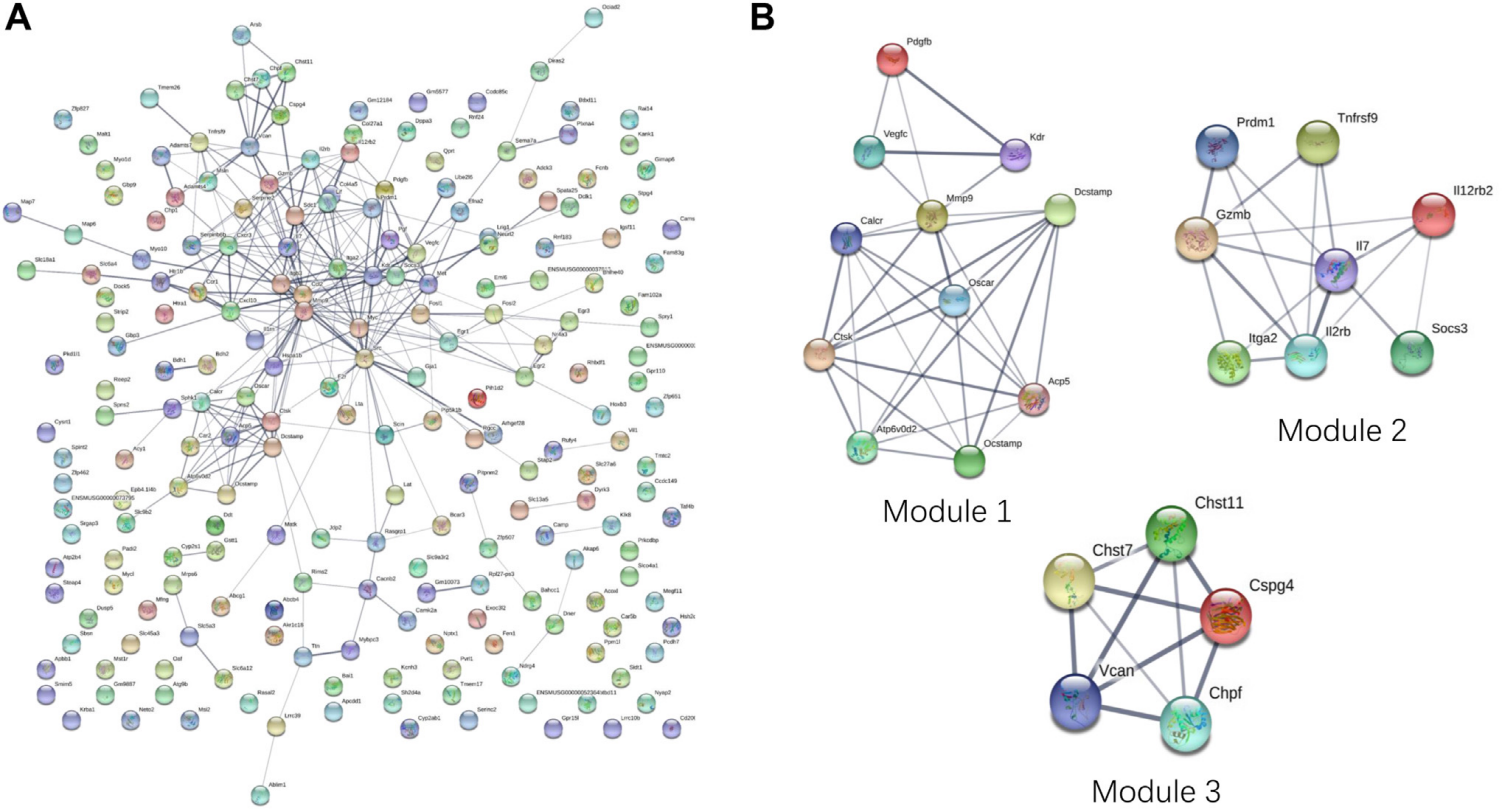
**Core interaction modules in the PPI of differentially expressed genes.***A*, the PPI diagram of downregulated genes, where nodes denoted genes and connecting lines indicated the existence of interactions between two genes. The more lines connected to a node, the greater its connectivity is, which indicates that the gene represented by that node is more important in the networks. *B*, the three modules with the highest connectivity in the networks were associated with osteoclast differentiation and bone resorption function. PPI, protein–protein interaction.

We then performed protein–protein interaction (PPI) network analysis on the corresponding differentially expressed genes. We found three core interaction modules in the interaction networks of the differentially expressed genes, and modules 1, 2, and 3 showed the core genes involved, respectively. Figure 7*A* presented the PPI of downregulated genes, where the nodes denoted genes, and the connecting lines indicated the existence of interactions between two genes. The more lines connected to a node, the higher its connectivity, indicating that the gene node is more important in the network. Figure 7*B* showed the three modules with the highest connectivity in the networks, all of which were associated with osteoclast differentiation and bone resorption function.

## Discussion

Osteoporosis is a disease that results from osteoclast dysfunction, the exact mechanism of which still remains elusive. The role of PKM2 in many pathophysiological processes has been described. However, its role in osteoclastogenesis remains unclear. Kim *et al.* (23) found that the immunoglobulin superfamily 11–PKM2 axis is involved in the regulation of glucose metabolism and affects osteoclast differentiation. Immunoglobulin superfamily 11 phosphorylates PKM2 at the Y105 site through various src family kinases, which inhibits PKM2 activity and modulates glycolysis in BMMs. Another study emphasized the importance of PKM2 in osteoclastogenesis *in vitro* and *in vivo* and speculated that PKM2 may regulate osteoclastogenesis by affecting signal transducer and activator of transcription 3, but the exact mechanism was not elucidated (24).

Interestingly, our study revealed that silencing PKM2 by shRNA (Figs. 1, *D*–*I* and 2) or its activators DASA-58 and TEPP-46 (Figs. 3, *C*–*I* and 4) in BMMs could both inhibit osteoclast differentiation, bone resorption function, and expression of osteoclast-associated genes, suggesting that PKM2 may play an important part in osteoclastogenesis, which was consistent with the previous findings (23, 24, 25). Contrary to the conventional perception, the knockdown of PKM2 has been found to have the same inhibitory effect on osteoclastogenesis as PKM2 activators. Since PKM2 activators could promote the conversion of PKM2 from monomeric or dimeric form to tetrameric form, and the tetramers were unable to enter the nucleus as transcription factors to promote transcription and gene expression as monomers or dimers do, we speculate that PKM2 activators, such as DASA-58 and TEPP-46, remain in the cytoplasm to exert enzymatic activities rather than transcriptional activities.

We further investigated the mechanism by which PKM2 regulated osteoclastogenesis and found that treatment with TEPP-46 or DASA-58 both inhibited the phosphorylation of the NF-κB and MAPK pathways (Fig. 5). Osteoclast differentiation and formation is a process in which monocytes fuse with each other to form multinucleated cells (26). During the differentiation of BMMs toward osteoclasts, monocytes fuse to form multinucleated cells, in which OC-STAMP and DC-STAMP play an important role (27). OC-STAMP and DC-STAMP have been shown to promote the fusion of osteoclast precursor cells and are the major regulatory proteins of osteoclastogenesis (26, 28, 29, 30). Recent studies have found that small molecule–mediated downregulation of DC-STAMP was involved in the inhibition of osteoclast differentiation and bone loss (31, 32, 33). They also regulate osteoclast and osteoblast differentiation to influence bone metabolic homeostasis (34, 35, 36). Interestingly, RNA-seq results showed a significant downregulation of OC-STAMP and DC-STAMP expression (Fig. 6, *A* and *B*). Knockdown of PKM2 or treatment with PKM2 activators, DASA-58 and TEPP-46, in BMMs both affected osteoclast precursor cell fusion by inhibiting OC-STAMP and DC-STAMP (Figs. 6, *C*–*F* and 7). Moreover, Mullin *et al.* (37) found that DC-STAMP gene had an established role in osteoclast differentiation using osteoclast-specific expression quantitative trait locus dataset and genome wide association study. Thus, we speculate that PKM2 regulated osteoclastogenesis by affecting the NF-κB and MAPK pathways through OC-STAMP and DC-STAMP.

There are some potential limitations to our study. We know little about the mechanisms by which the small-molecule activators, DASA-58 and TEPP-46, inhibit osteoclastogenesis. DASA-58 and TEPP-46 could promote the formation of PKM2 tetramer and inhibit its entry into the nucleus while inhibiting the activity of PKM2. However, it needs to be further investigated whether this effect could have an inhibitory effect on osteoclast differentiation. Furthermore, when we treated BMMs with the PKM2 activators, DASA-58 and TEPP-46, the expression of OC-STAMP and DC-STAMP was inhibited, but it is still unclear whether this inhibition is a direct effect or because of other factors at the transcriptional level, which requires further studies to verify. Although we have demonstrated the role of knockdown of PKM2 and PKM2 activators in osteoclast differentiation *in vitro*, their role *in vivo* requires further investigation.

In conclusion, PKM2 may be a new target for intervention in osteoclasts and play a prominent role in inhibiting osteoclast differentiation *via* downregulation of OC-STAMP and DC-STAMP, providing a new therapeutic strategy for diseases associated with osteoclast lesions.

## Experimental procedures

### Reagents

DASA-58 and TEPP-46 were purchased from Selleck. Recombinant soluble murine M-CSF and RANKL were purchased from PeproTech. PBS, fetal bovine serum, and Dulbecco's modified Eagle's medium were purchased from Thermo Fisher Scientific. TRIzol reagent was purchased from Invitrogen. MTT cell proliferation and cytotoxicity assay kit was purchased from Boster Biotechnology. The following antibodies were purchased from Cell Signaling Technology: GAPDH (#2118), ERK (#9102), phospho-ERK (#4377), JNK (#9258), phospho-JNK (#4668), P38 (#8690), phospho-P38 (#4511), IKKβ (#8943), phospho-IKKα/β (#2697), P65 (#8242), phospho-P65 (#3033), IκB-α (#4812), phospho-IκB-α (#2859), and NFATc1 (#8032). Anti-TRAP antibody (#185716) was purchased from Abcam. Antibodies against CTSK (#11239-1-AP) and PKM2 (#15822-1-AP) were purchased from Proteintech Group. Antibody against DC-STAMP (#A14630) was obtained from ABclonal Technology. The Osteo Assay Surface Polystyrene 1 × 8 Stripwell Microplate (No. 3989) was purchased from Corning Life Sciences. The TRAP staining kit and all other reagents were purchased from Sigma–Aldrich.

### Cell isolation, culture, and differentiation

BMMs were obtained from 6- to 8-week-old C57BL/6 male mice, provided by the Animal Experiment Center of China Three Gorges University. Mice were executed under carbon dioxide anesthesia followed by immersion in alcohol for 15 min, and the femur and tibia of both hindlimbs were isolated. Dulbecco's modified Eagle's culture medium was aspirated with a 5 ml syringe, and the femoral and tibial bone marrow was rinsed three times into sterile culture dishes. The cell culture medium in the culture dish was made up to 10 ml with M-CSF at a concentration of 25 ng/ml and then incubated in a cell incubator containing 5% CO_2_ at 37 °C for 24 h. The supernatant in the culture dish was collected, transferred to a new culture dish, supplemented with 3 ml of culture medium and the corresponding amount of M-CSF, and then incubated in a cell incubator with 5% CO_2_ at 37 °C for another 24 h. Afterward, 3 ml of culture medium and the corresponding amount of M-CSF were added, and the cells were incubated for 24 h under the same conditions. The medium was then changed, and the appropriate amount of M-CSF was added and cells were incubated for another 24 h under the same circumstances. After digestion with trypsin for 2 min, BMMs were gently blown to resuspend and then inoculated into culture plates for subsequent experiments. RANKL was added at a concentration of 100 ng/ml to induce differentiation of BMMs toward osteoclasts. The culture medium was changed daily.

### Adenovirus transfection

Adenovirus carrying shRNA targeting murine PKM2 (NM_011099) and control viruses were cloned and packaged by Vigene Biosciences. Four shRNAs were designed in one vector, targeting different regions of PKM2. The sequences for each shRNA are listed in Table 1.

**Table 1:** The sequences for each shRNA of PKM2

shRNA segment	Sequences (5′-3′)
shRNA1	GACATGGTGTTTGCATCTTTCTTCAAGAGAGAAAGATGCAAACACCATGTCTTTTTT
shRNA2	GCAGGTTTGATGAGATCTTGGTTCAAGAGACCAAGATCTCATCAAACCTGCTTTTTT
shRNA3	GAGATGCTGAAGGAGATGATTATTCAAGAGATAATCATCTCCTTCAGCATCTTTTTTT
shRNA4	GATCATTGCCGTGACTCGAAATTTCAAGAGAATTTCGAGTCACGGCAATGATTTTTTT

### Cytotoxicity and cell proliferation assay

BMMs were seeded at a density of 3 × 10^3^ cells/well in 96-well plates for the cytotoxicity assay. After 24 h, cells were treated with various concentrations of the PKM2 activators, DASA-58 and TEPP-46 (13, 38, 39, 40, 41, 42). After 1, 3, and 5 days of incubation, the absorbance was measured at 450 nm using a microplate reader (BioTek).

### TRAP staining

To examine the effects of knockdown of PKM2 and PKM2 activators, DASA-58 and TEPP-46, on osteoclast formation in BMMs, cells were treated with DASA-58 and TEPP-46 at the indicated concentrations for 6 days. The TRAP staining kit was used to identify osteoclasts according to the manufacturer's instructions; mature osteoclasts were identified as TRAP-positive multinucleated cells with three or more nuclei (43). Images were captured using an inverted fluorescence microscope (EVOS FL Auto; LifeTechnologies).

### Cell morphology

BMMs were treated with M-CSF (25 ng/ml), RANKL (50 ng/ml), and different concentrations of PKM2 activators, DASA-58 and TEPP-46, for 5 days to form mature osteoclasts. Cells were fixed with 4% paraformaldehyde for 15 min, followed by permeabilization with 0.1% Triton X-100 for 5 min, and then incubated with TRITC-Phalloidin or FITC-Phalloidin (Sigma–Aldrich) for 30 min at room temperature to visualize F-actin. Cells were then washed three times with PBS, and 4',6-diamidino-2-phenylindole (Yeasen) was added for 5 min. Images were captured using an inverted fluorescence microscope (EVOS FL Auto).

### Pit formation assay

Transfected and untransfected BMMs (10 × 10^3^ cells/well) were cultured in a 6-well plate with M-CSF (25 ng/ml) and RANKL (50 ng/ml) for 5 days. Cells were digested with trypsin and seeded into a 96-well Corning Osteo Assay Surface plate and then treated with M-CSF (25 ng/ml) and RANKL (50 ng/ml) for 4 days. Cells were rinsed three times with a bleach solution, and the absorption area was measured for quantitative assessment of pit formation.

### Quantitative real-time RT–qPCR

Total RNA was extracted, reverse transcribed to complementary DNA (cDNA), and RT–qPCR was performed to detect the expression of osteoclast-related genes. Briefly, total RNA was isolated from cultured BMMs using TRIzol reagents according to the manufacturer's instructions, and cDNA was then synthesized with Hifair Ⅲ first Strand cDNA Synthesis SuperMix for qPCR (gDNA digester plus; Yeasen). Templates were amplified with Hieff qPCR SYBR Green Master Mix (No Rox; Yeasen) on CFX Connect Real-Time PCR Detection System (Bio-Rad). Primers synthesized by Tsingke were used as shown in Table 2. The relative mRNA levels of genes were calculated by the 2^−ΔΔCt^ method using GAPDH as an internal control and normalized to the control.

**Table 2:** Primer sequences used in RT–qPCR

Gene	Forward primer sequence (5′-3′)	Reverse primer sequence (3′-5′)
*RANKL*	CAGGAGAGGCATTATGAGCA	GGTACTTTCCTGGTTCGCAT
*TRAP*	GATGCCAGCGACAAGAGGTT	CATACCAGGGGATGTTGCGAA
*CTSK*	GAAGAAGACTCACCAGAAGCAG	TCCAGGTTATGGGCAGAGATT
*MMP-9*	CTGGACAGCCAGACACTAAAG	CTCGCGGCAAGTCTTCAGAG
*c-Fos*	GGTGAAGAGCCGTGTCAGGAG	TATTCCGTTCCCTTCGGATT
*NFATc1*	CAACGCCCTGACCACCGATAG	GGGAAGTCAGAAGTGGGTGGA
*GAPDH*	ACCCAGAAGACTGTGGATGG	CACATTGGGGGTAGGAACAC

### Western blotting

Cells were washed twice in prechilled PBS, and 150 μl of enhanced RIPA lysate (Boster Biotechnology) containing 1 mM PMSF was added to each well and lysed on ice for 15 min. The cell lysate was scraped off with a clean cell scraper, carefully aspirated with a pipet tip into a clean 1.5 ml EP tube, and centrifuged at 4°C for 20 min at 12,000 rpm. The supernatant was then carefully aspirated with a pipet tip, and protein concentration was determined using BCA Protein Assay Kit (Beyotime) according to the instructions. Western blotting was then performed according to the protocol from Cell Signaling Technology. Afterward, the bands were captured by a molecular imager (ChemiDoc XRS+; Bio-Rad) and analyzed by ImageLab software (Bio-Rad). All the experiments were repeated at least three times.

### RNA-seq

BMMs (50 × 10^4^ cells/well) were inoculated in a 6-well plate. The experimental group was treated with DASA-48 and RANKL, and the control group was treated with an equal volume of DMSO. Total RNA was extracted for RNA-seq after 3 days, with three replicates for each group. Gene expression profiles were detected by Annoroad Gene Technology Corp. Differentially downregulated genes were defined as genes with log fold change <−1 and *p* < 0.05 and were presented in a heatmap. Gene function enrichment analysis was performed using R (version 3.6.2). PPI network analysis of the differentially downregulated genes was performed using online tools at www.string-db.org, and the module analysis was performed in Cytoscape (version 3.7.1).

### Statistical analysis

Each experiment was replicated at least three times. SPSS 19.0 (IBM) was used to process the experimental data, and all data were expressed as mean ± standard deviation. Student's *t* test for independent samples was used to compare two groups of experimental data, and the one-way ANOVA test was used for measurement data of more than two groups. Results were considered statistically significant when *p* < 0.05.

## Data availability

The RNA-seq expression data analyzed for this study are available in the Gene Expression Omnibus database (http://www.ncbi.nlm.nih.gov/geo/), accession number GSE188963. There are no restrictions on the availability of the data after publication of the article.

## Conflict of interest

The authors declare that they have no conflicts of interest with the contents of this article. The authors declare that the research was conducted in the absence of any commercial or financial relationships that could be construed as a potential conflict of interest.
